# A Tribute to Doctors

**Published:** 1906-03

**Authors:** Carrie Belle Sterrett


For Texas Medical Journal.

### A Tribute to Doctors.

BY CARRIE BELLE STERRETT.*

It is a great thing to have a profession, but it is still greater to belong to that one of which Christ was a charter member.

To the one who really lives, the object of life is service; and he serves best who thinks of self least. To what profession is this more applicable or more true than that of the physician? Is there any one more ready to impart the knowledge he has slaved for? Who is it who gives up the comforts of life more than the doctor? You have seen pictures of "The man with the hoe," "The Lark," "The Sower," and many others which appealed to you as works of art, but did they awaken in you that emotion caused by admiration and sympathy for the man who does an unselfish, brave act, as does the famous picture of "The Country Doctor"? Unselfishness and love for others is written in its every line.

The "Great Physician" has said: "Greater love hath no man than this,—that a man lay down his life for his friend's."

What is it that prompts a doctor to get up out of a warm bed at night, ford streams, and brave the storm? Is it the thought of money? No, most emphatically, no. The sufferer is apt to be one who he knows can never pay a cent. What is it, then? We answer with all seriousness, "love." No man better carries out the commandment, "Love thy neighbor," than the doctor.

Ian McLaren, in the "Bonnie Brier Bush," beautifully portrays the sympathy that binds the doctor to his patients. The little story goes something like this: Annie, the wife of Tammas, lay at death's door, and when Tammas begged Dr. McClure to save her, the old doctor answered, "Ye need na plead wi me, Tammas, to dae the best I can for yer wife. Man, I kent her lang afore ye ever loved her; I brocht her intae the world and I saw her through her fever when she was a bit lassikie; I closed her mither's een, and it was me hed tae tell her she was an orphan, and nae man was better pleased when she got a gude husband. I've naither wife nor bairns of my own, an' I count a' the foulk of the Glen ma family. Dae ye think I wudna save Annie if I could? Tam-

*Miss Carrie is the young daughter of Dr. M. D. Sterrett, of Beckville, Texas, a distinguished ex-Confederate surgeon. She is a junior student at the University of Texas. Her little essay received the highest grading in its class. She permits me to use it at my special request.—Dr. Daniel.

mas, my puir fellow, if it could avail, I tell ye I would lay doon this auld worn-out wickle o' a body o' mine just tae see ye baith sittin' at the fireside, and the bairns round ye, courthy and canty again; but it's to nae be, Tammas, it's nae to be." Just as Dr. McClure counted all the people at the Glen his family, so every doctor thinks of himself as the servant of mankind, and his sympathies are with the world.

The average doctor is poor in purse, but rich in memories of duty done, and his desire to do good. Wealth does not mean an elegant home, fine clothes, and money in the bank. He is wealthiest whose friends recall his past as a beautiful, soul-inspiring picture. Of what physician is this not true?

The doctor's profession is higher than that of a preacher. The Christian doctor not only has the power to minister to his patients physically, but spiritually, as well. When Christ was on this earth, he first attended to the human needs before he attempted to convince them of spiritual needs. So the doctor's duty is twofold.

Others may have monuments built to their memory; others may win the plaudits of the multitude; but not so with the humble, faithful doctor. Only in the still hour of night, by the lone couch of suffering, when the eyes of the world are closed and its ears hear not, only there, when with tears, the sorrows, and gratitude of helpless ones, does the doctor receive his earthly reward. There comes a time, though, when he is overcome at last in the struggle for others, and he goes to wear the heavenly crown, begemmed and sanctified by good deeds. What higher reward can be given?

